# Three-dimensional printed upper-limb prostheses lack randomised controlled trials: A systematic review

**DOI:** 10.1177/0309364617704803

**Published:** 2017-06-24

**Authors:** Laura E Diment, Mark S Thompson, Jeroen HM Bergmann

**Affiliations:** Department of Engineering Science, University of Oxford, Oxford, UK

**Keywords:** Computer-aided design–computer-aided manufacturing, rapid prototyping, upper-limb prosthetics, prosthetic design, children’s prosthetics, evaluation studies, study design

## Abstract

**Background:**

Three-dimensional printing provides an exciting opportunity to customise upper-limb prostheses.

**Objective:**

This review summarises the research that assesses the efficacy and effectiveness of three-dimensional printed upper-limb prostheses.

**Study design:**

Systematic review.

**Methods:**

PubMed, Web of Science and OVID were systematically searched for studies that reported human trials of three-dimensional printed upper-limb prostheses. The studies matching the language, peer-review and relevance criteria were ranked by level of evidence and critically appraised using the Downs and Black Quality Index.

**Results:**

After removing duplicates, 321 records were identified. Eight papers met the inclusion criteria. No studies used controls; five were case studies and three were small case-series studies. All studies showed promising results, but none demonstrated external validity, avoidance of bias or statistically significant improvements over conventional prostheses. The studies demonstrated proof-of-concept rather than assessing efficacy, and the devices were designed to prioritise reduction of manufacturing costs, not customisability for comfort and function.

**Conclusion:**

The potential of three-dimensional printing for individual customisation has yet to be fully realised, and the efficacy and effectiveness to be rigorously assessed. Until randomised controlled trials with follow-up are performed, the comfort, functionality, durability and long-term effects on quality of life remain unknown.

**Clinical relevance:**

Initial studies suggest that three-dimensional printing shows promise for customising low-cost upper-limb prosthetics. However, the efficacy and effectiveness of these devices have yet to be rigorously assessed. Until randomised controlled trials with follow-up are performed, the comfort, functionality, durability and long-term effects on patient quality of life remain unknown.

## Background

Additive manufacturing, more commonly referred to as three-dimensional printing (3DP), provides an exciting opportunity to create custom-made low-cost prosthetic upper limbs. The manufacturing method prints layer-by-layer from a digital 3D model. 3DP has been used for decades to make non-functional prototypes, but advances in process capabilities and materials have enabled finished components to be printed directly from software.^1,2^ It is easy and inexpensive to customise each print, and it is less labour intensive than manufacturing traditional prostheses. Furthermore, open-source databases (e.g. E-Nable, a non-profit organisation^3^ and Open Bionics, a for-profit organisation^4^) of body-powered and bionic 3DP designs are readily available for printing on consumer printers. These allow amputees to modify, print and trial their own devices without requiring any specialist equipment.

3DP technology supports a data-driven approach, allowing additional information from sensors, previous designs and machine-learning to be incorporated into the devices, potentially reducing trial-and-error in fitting. With more than 100 million people globally in need of a prosthesis or orthosis, 3DP may also help increase access above current figures of 1 in 10.^5^

For a new therapy to be accepted by healthcare regulatory bodies or recommended for reimbursement by insurance companies, its efficacy and effectiveness must be shown. Efficacy is defined as the performance of the device under ideal and controlled conditions, and effectiveness is defined as its performance under everyday conditions.^6^ The Food and Drug Administration (FDA) states that 3DP medical devices are required to meet the same regulations as their non-3DP counterparts and expects 3DP devices to rarely raise different questions on safety and effectiveness.^7^ Evidence of efficacy and effectiveness for medical devices requires randomised clinical trials, which are published in peer-reviewed journals.

In reviewing the relevant literature, the level of evidence^8^ and the quality of the study can be assessed using published tools.^9^ In particular, this determines whether the reported trial is designed to ask an appropriate question that relates to how the device is to be used – that is, ‘external validity’ – and whether the study answers the research question in an unbiased way – that is, ‘internal validity’.^10^

Despite the growing popularity of 3DP upper-limb prosthetics, no review of their efficacy and effectiveness has been carried out. This review aims to assess the evidence for the clinical efficacy and effectiveness of 3DP upper-limb devices by performing a systematic literature search, categorising studies by their level of evidence, and critically appraising their scientific quality.

## Methods

A systematic review of literature was performed using PubMed, Web of Science and OVID to find manuscripts that reported human trials of 3D printed upper-limb prosthetics. The review follows the Preferred Reporting Items for Systematic Reviews and Meta-Analyses (PRISMA) guidelines.^11^ This systematic review does not require ethical approval.

The 73 terms included in the search covered areas under three MESH headings: ‘health care evaluation mechanisms’, ‘printing, three-dimensional’ and ‘prostheses and implants’. The search includes all publications up to September 2016 (see Table 1 for full search strategy).

**Table 1.** The search terms with the MESH headings in bold.

‘**health care evaluation mechanisms**’ OR ‘randomized controlled’ OR ‘randomised controlled’ OR RCT OR ‘randomized control’ OR ‘randomised control’ OR ‘clinical trial’ OR ‘clinical study’ or ‘clinical evaluation’ OR ‘case-control’ or ‘case control’ OR ‘case study’ OR ‘case report’ OR ‘pilot study’ OR efficacy OR effectiveness OR evaluation OR validation OR feasibility OR cohort OR observational OR longitudinal OR retrospective OR comparative OR exploratory OR subjects OR participant OR participants OR patient OR patients AND (‘**Printing, Three-Dimensional**’ OR (‘three dimensional’ OR ‘Three-dimensional’ OR ‘3 Dimensional’ OR 3D OR ‘3-D’ AND (printing OR print OR printed)) OR ‘3D-printing’ OR ‘3D-print’ OR ‘3D-printed’ OR stereolithography OR ‘laser melting’ OR ‘electron beam melting’ OR ‘fused deposition modeling’ OR ‘fused deposition modelling’ OR ‘selective laser sintering’ OR ‘rapid manufacturing’ OR ‘rapid prototyping’ OR ‘layered manufacturing’ OR ‘digital manufacturing’ OR ‘3D prototyping’ OR ‘3D fabrication’ OR ‘rapid fabrication’ OR ‘freeform fabrication’) AND (‘**prostheses and implants**’ OR prosthetic OR prosthetics OR prosthesis OR prostheses OR artificial OR bionic OR robotic AND finger OR hand OR wrist OR transradial or ‘trans-radial’ or transhumeral or ‘trans-humeral’ OR shoulder OR ‘upper-limb’ OR ‘upper limb’ OR ‘upper-extremity’ OR ‘upper extremity’)

The title and abstract of each publication was reviewed to assess whether it met the inclusion criteria of:

1. *Relevance*. Manuscripts were required to report on a trial with human participants that tested a 3D printed upper-limb prosthesis.
2. *Language*. The review only included manuscripts written or translated into English.
3. *Peer-review*. Manuscripts were required to have been through a peer-review process.

A full-text review of the included studies was undertaken and any cited references that met the inclusion criteria or papers suggested by experts were added. The studies were then rated according to their level of evidence, based on the Oxford Centre for Evidence-based Medicine (CEBM) Levels of Evidence.^8^ The Downs and Black^9^ Quality Index was used to critically analyse the studies under review. It has 27 questions divided into ‘reporting’, ‘external validity’, ‘internal validity’ both in regards to bias and confounding, and ‘power’. Instead of assigning weights to the items in the scale, the responses have been left in their raw form (yes/no/unable to determine/not applicable) and presented in a table to enable the reader to visualise the trends, as recommended by the Cochrane Handbook for Systematic Reviews of Interventions.^10^ A summary of the included studies (intervention, details of participants and primary outcomes) was also tabulated.

## Results

**Figure 1.**
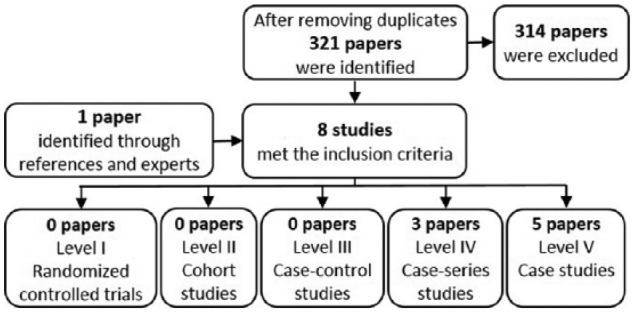
Flow chart of the selection and sorting method.

After removing duplicates, 321 records were identified. Of these, seven met the relevance, language and peer-review inclusion criteria (Figure 1). The references cited in the included studies did not reveal any further studies that met the inclusion criteria but one additional study was found by a field expert and included. The studies were then rated according to their level of evidence. All eight were small non-randomised trials without controls, five of which were individual case studies (Figure 1).

The eight studies evaluated are summarised in Table 2. Only one was an above-elbow device. The remaining seven studies included hands, a finger actuator and an exoskeleton for arm rehabilitation that was labelled as an exoprosthesis. One study was designed to assess whether it is feasible to fit a prosthesis from a distance,^17^ and another used the same prosthesis design but focused on assessing the physical changes that occurred in the participant after 6 months of use. The other six studies aimed to assess the functionality of the device, but the focus of the papers was on describing the design of the prosthesis rather than assessing its clinical efficacy and effectiveness. Of the five studies that tested a device with amputees, four were tested with children. The other devices have not been clinically trialled with amputees.

**Table 2.** Summary of studies that assess the efficacy and effectiveness of a 3DP upper-limb device.

Author	Device	Participants	Aim	Outcomes
Gretsch et al.^12^	Robotic below-elbow prosthesis	1 amputee Age: 13 Gender: female	To design and test an inexpensive, externally powered 3DP prosthesis that does not require a functional wrist	The patient said that the biggest advantages were the individual thumb movement, the ability to grasp objects with all fingers and the low weight compared to other externally powered prostheses
Kontoudis et al.^13^	Robotic hand	1 able-bodied participant Age and gender not provided	To test the efficacy of the hand design through experimental paradigms involving gestures and grasping of objects	The fingers could move independently using a single motor, and the user was able to grasp objects of varied sizes and shapes
Low et al.^14^	Soft pneumatic finger actuator	5 able-bodied participants Age: 25 ± 2.5 Gender: 3 males, 2 females	To evaluate the capability of the soft actuators to grasp and hold different sizes and weights of objects	The prototype was able to grasp the four items firmly, move them in all three axis directions and rotate the objects 90°
Sorin et al.^15^	3DP gears used in a myoelectric below-elbow exoskeleton for the arm	1 low strength participant Age: 31 Gender: male	To compare the movements of the arm in the exoskeleton to those of the natural arm	The exoskeleton caused the affected arm to move similarly to the natural arm
Yoshikawa et al.^16^	Electric prosthetic hand	1 amputee Age: 40s Gender: male	To assess whether the Rehand enabled an amputee to do everyday activities, using the Southampton Hand Assessment Procedure	All light-object tasks were completed in 100 s. For heavy-object tasks, the sphere task and the lateral task were unable to be completed. Most Activities of Daily Living tasks were completed, but those that required fine manipulation were not
Zuniga et al.^17^	Child’s wrist activated voluntary-closing hand	11 amputees Age: 3–16 Gender: 7 males, 2 females, 2 information not provided	To test a prosthesis fitting methodology that can be performed at a distance	No significant mean differences were found between the anthropometric and range-of-motion measurements taken directly from the upper limbs of subjects versus those extracted from photographs
Zuniga et al.^18^	Child’s wrist activated voluntary-closing hand	5 amputees Age: 3–10 Gender: 3 males, 2 females	To identify anthropometric, range of motion (ROM) and strength changes after using the prosthesis for 6 months	Forearm circumference increased (*p* = 0.02), as did ROM for flexion (*p* = 0.02) and extension (*p* = 0.04). No significant change was found for wrist flexion or extension strength or ROM for ulnar or radial deviation (*p* > 0.05)
Zuniga et al.^19^	Mechanical shoulder prosthesis	1 amputee Age: 7 Gender: male	To test whether an inexpensive 3DP mechanical shoulder prosthesis could assist an amputee in performing bimanual activities	A partial correction of the patient’s spinal deviation was noted due to the counterweight of the device. The family reported improved balance and performance of some bimanual activities after 2 weeks of use

3DP: three-dimensional printing.

A critical appraisal of the studies shows that no studies compared the 3DP device to current products or methods, blinded the participants or those measuring the outcomes to the intervention, or demonstrated external validity (Table 3, with abridged Quality Index questions). Three studies included follow-up; one followed up the patient after 2 weeks of prosthesis use, another between 1 and 3 months after first use and the third after 6 months of use. Despite all papers reporting positive outcomes of using the 3DP devices, only one had sufficient power to detect a clinically important effect. This one showed increase in wrist range of motion and wrist circumference after 6 months of use.

**Table 3.** Critical appraisal of studies using the Downs and Black9 Quality Index.

+	Yes	**First author, date**	Gretsch, 2016	Kontoudis, 2015	Low, 2015	Sorin, 2015	Yoshikawa, 2015	Zuniga, 2015	Zuniga, 2016a	Zuniga, 2016b
−	No									
?	Unsure									
x	Not applicable									
**Level of evidence**			5	5	4	5	5	4	4	5
1. Clear hypothesis/aim/objective		**Reporting**	+	+	+	+	−	+	+	+
2. Clear outcome measures			−	−	−	−	+	+	+	−
3. Patient characteristics described			+	−	+	+	+	+	+	+
4. Interventions clearly described			−	+	+	+	+	+	+	−
5. Distributions of confounders described			x	x	x	x	x	x	x	x
6. Findings clearly described			−	−	−	−	+	+	+	−
7. Estimates given of random variability			−	−	−	−	−	+	+	−
8. Adverse events reported			−	−	−	−	−	−	+	−
9. Patients lost to follow-up described			x	x	x	x	x	+	+	+
10. Probability values reported			−	−	−	−	−	−	+	−
11. Recruitment pool represents population		**External validity**	x	−	−	−	x	?	?	x
12. Participants represent population			x	−	−	−	x	?	?	x
13. Staff/places/facilities match std. treatment			−	−	−	−	?	?	−	−
14. Participants blinded to intervention		**Internal validity - bias**	−	−	−	−	−	−	−	−
15. Those measuring outcomes blinded			−	−	−	−	−	−	−	−
16. Data dredging reported			x	x	+	x	+	+	+	x
17. Adjusted for different lengths of follow-up			x	x	x	x	x	x	+	x
18. Appropriate statistical tests			x	x	x	x	x	+	+	x
19. Reliable compliance with intervention			?	+	+	+	+	?	?	?
20. Accurate/reliable outcome measures			x	?	−	?	+	+	+	?
21. Groups recruited from same population		**Internal validity - confounding**	x	x	x	x	x	x	x	x
22. Groups recruited over same timeframe			x	x	x	x	x	x	x	x
23. Subjects randomised into intervention			x	x	x	x	x	x	x	x
24. Randomised intervention concealed			x	x	x	x	x	x	x	x
25. Adjustment for confounding			x	x	x	x	x	x	x	x
26. Losses to follow-up accounted for			x	x	x	x	x	+	+	+
27. Sufficient power		**Power**	−	−	−	−	−	−	+	−

## Discussion

The results of this review show that no studies have appropriately assessed the efficacy or effectiveness of using additive manufacturing for upper-limb prosthetics. None of the studies used a control group to evaluate the effects of using the 3DP device or demonstrated an improvement over conventional prostheses. However, one study showed that a 3DP hand significantly improved wrist range of motion and circumference after 6 months of use. The case studies and pilot studies that were found demonstrated limited internal validity and did not demonstrate external validity. Therefore, the results and conclusions of the studies must be used with caution.

Outcomes reported were mainly on 3DP device kinematics, especially of the terminal device, and qualitative functional assessment was highly positive. Three studies used grasping tasks for functional assessment, but only one used a validated test procedure.^16^ It showed that the user was unable to grip heavier items or complete tasks that required fine manipulation. One study showed no significant mean difference between anthropometric measures taken on the subject’s arm and those taken from photographs, suggesting that there may be potential for remote fitting of 3DP prostheses. However, they made no assessment of the outcomes of fitting provided in this way.^17^

None of the reports assessed 3DP device strength and durability, though one study indicated that three out of five participants reported breaking or malfunctioning of the prosthesis over a 6-month period.^18^ 3DP material is highly anisotropic, with different mechanical properties depending on loading direction, and prosthetics are subjected to complex, cyclic loading. The strength of 3DP is typically lower than an injection-moulded equivalent.^20^ 3DP materials and designs need to be carefully chosen in order to ensure that they have the necessary strength and durability to support everyday use. Only one report of an above-elbow prosthesis was found,^19^ potentially reflecting the difficulty of meeting even basic strength requirements. No follow-up longer than 6 months was reported, so there is limited information on durability and wear of the devices.

A key benefit to using 3DP for medical applications is its ability to print devices customised to the patient. Four studies customised the size of device for the participant and two others discussed the possibility of custom sizing. However, research into using 3DP to support a data-driven approach to customisation, such as feeding information from scans or sensors into the digital model to improve comfort or to match the limb shape to the contralateral one, has yet to be explored. In the field of lower-limb prosthetics, there has been preliminary research into using magnetic resonance imaging (MRI) or computed tomography (CT) data to assess bone depth so as to print cushioned areas into the socket using a multi-material 3D printer.^21^ This research may be transferable to upper-limb prosthetics. The main reported advantages of 3DP from the upper-limb studies were low manufacturing costs for small print runs, ease-of-access for the researchers and options for making the design open-source.

Non-trained professionals frequently provide prosthetics services, often resulting in poor fit, inadequate rehabilitation support and secondary complications.^5^ There is a danger that users who self-print their device may not receive the support they require to maximise their function.^22^ Therefore, the implementation of any 3DP prosthetics service will require careful design and monitoring.

In 2016, the FDA released draft guidance on the technical considerations in 3DP.^7^ There remains ambiguity in how non-traditional manufacturers, such as hospitals and clinics, must adhere to these regulations and how these sites will be regulated. However, as further clarity is provided on these issues, it is expected that these manufacturers will also be required to demonstrate proof of device efficacy and effectiveness before prescribing 3DP devices for patients.^6,7^

Providing a critical appraisal of the efficacy and effectiveness of 3DP upper-limb devices gives healthcare professionals a resource to assess the validity of the devices and provides researchers with an overview of areas that require further research and validation. Comparing a device to current devices and procedures and understanding the long-term implications of the device’s use is important for validating the device and insuring best patient outcomes. The studies in this review mainly focused on describing the designs and providing a limited assessment of their functionality rather than assessing the devices’ clinical efficacy and effectiveness. No papers presented randomised or controlled trials, and there was not enough data to perform a meta-analysis to assess the efficacy of 3DP for upper-limb prosthetics. It is therefore imperative that before 3DP prostheses are widely promoted and distributed, randomised controlled trials of the efficacy and effectiveness of using 3D printed devices are performed with long-term follow-up.

## Conclusion

3DP provides exciting new opportunities for the field of upper-limb prosthetics, but much more research and validation is required before the many potential benefits can be realised in clinical practice. Thus far, there is limited evidence of the efficacy or effectiveness of using 3DP upper-limb prostheses, with few studies reporting the outcomes of trialling the devices with amputees. Although all eight studies showed promising results, the studies were small, lacked internal and external validity, and did not use controls. The authors of the review share the optimism and enthusiasm for 3DP prosthetics but recommend that randomised controlled clinical trials are performed to assess the efficacy and effectiveness of 3DP prostheses before integrating 3DP upper-limb devices into standard clinical practice.
